# High body mass index is a significant risk factor for the progression and prognosis of imported COVID-19: a multicenter, retrospective cohort study

**DOI:** 10.1186/s12879-021-05818-0

**Published:** 2021-02-05

**Authors:** Huan Cai, Lisha Yang, Yingfeng Lu, Shanyan Zhang, Chanyuan Ye, Xiaoli Zhang, Guodong Yu, Jueqing Gu, Jiangshan Lian, Shaorui Hao, Jianhua Hu, Yimin Zhang, Ciliang Jin, Jifang Sheng, Yida Yang, Hongyu Jia

**Affiliations:** State Key Laboratory for Diagnosis and Treatment of Infectious Diseases, National Clinical Research Center for Infectious Diseases, Collaborative Innovation Center for Diagnosis and Treatment of Infectious Diseases, Department of Infectious Diseases, The First Affiliated Hospital, Zhejiang University of Medicine, 79 Qingchun Rd, Hangzhou, China

**Keywords:** High BMI, COVID-19, Risk factor, Progression, Prognosis

## Abstract

**Background:**

Coronavirus disease 2019(COVID-19) has spread worldwide. The present study aimed to characterize the clinical features and outcomes of imported COVID-19 patients with high body mass index (BMI) and the independent association of BMI with disease severity.

**Methods:**

In this retrospective cohort study, 455 imported COVID-19 patients were admitted and discharged in Zhejiang province by February 28, 2020. Epidemiological, demographic, clinical, laboratory, radiological, treatment, and outcome data were collected, analyzed and compared between patients with BMI ≥ 24and < 24.

**Results:**

A total of 268 patients had BMI < 24, and 187 patients had BMI ≥ 24. Those with high BMI were mostly men, had a smoking history, fever, cough, and sputum than those with BMI < 24. A large number of patients with BMI ≥ 24 were diagnosed as severe/critical types. Some biochemical indicators were significantly elevated in patients with BMI ≥ 24. Also, acute liver injury was the most common complication in these patients. The median days from illness onset to severe acute respiratory syndrome coronavirus 2 detection, duration of hospitalization, and days from illness onset to discharge were significantly longer in patients with BMI ≥ 24 than those with BMI < 24. High BMI, exposure to Wuhan, any coexisting medical condition, high temperature, C-reactive protein (CRP), and increased lactate dehydrogenase (LDH) were independent risk factors for severe/critical COVID-19. After adjusting for age, sex and above factors, BMI was still independently associated with progression to severe/critical illness (*P* = 0.0040). Hemoglobin, alanine aminotransferase (ALT), CRP, and serum creatinine (Scr) were independent risk factors associated with high BMI.

**Conclusions:**

Contrasted with the imported COVID-19 patients with BMI < 24, high proportion of COVID-19 patients with BMI ≥ 24 in our study, especially those with elevated CRP and LDH, developed to severe type, with longer hospitalization duration and anti-virus course. Thus, high BMI is a risk factor for the progression and prognosis of imported COVID-19.

**Supplementary Information:**

The online version contains supplementary material available at 10.1186/s12879-021-05818-0.

## Background

Coronavirus disease 2019 (COVID-19) has brought a major social and medical challenge for the whole world since the end of December 2019 [1–5]. A novel coronavirus, officially named as severe acute respiratory syndrome coronavirus 2 (SARS-CoV-2), was identified as the pathogen of COVID-19, which causes acute respiratory distress syndrome (ARDS), multi-organ failure, and other severe complications [4, 6]. By January 17, 2021, over 93 million cases were confirmed, and 2.0 million patients died worldwide due to the disease. Moreover, a total of 23,334,423 cases were confirmed and 389,084 patients died in the United States [7]. Therefore, undoubtedly, COVID-19 has become a major global public health threat.

Angiotensin-converting enzyme 2 (ACE2) was identified as the surface receptor for SARS-CoV-2 [8]. ACE2 is widely expressed in various organs and tissues, including lungs, cardiovascular system, kidneys, gut, bladder and brain [9–12], which might explain the multiple organ failure in some COVID-19 patients. Surprisingly, a recent study suggested that ACE2 expression in adipose tissue was higher than that in lung tissue [13]. Notably, no difference was detected in the level of ACE2 expression in adipocytes and adipose progenitor cells between obese and non-obese individuals [14]. However, since obese individuals have abundant adipose tissue to express a larger amount of ACE2 proteins may expose them to higher risk status for COVID-19 [13]. Furthermore, adipose tissue serves as a reservoir for influenza A virus, human immunodeficiency virus (HIV), cytomegalovirus, human adenovirus Ad-36, *Trypanosoma gondii*, and *Mycobacterium tuberculosis* [15]. Therefore, COVID-19 might also infect adipose tissue and then spread to other organs.

The number of obese people worldwide has tripled since 1975. In 2016, more than 1.9 billion adults > 18- year-old were overweight, among which over 650 million were obese [16]. Overweight/obesity is well acknowledged as a risk factor for increased mortality due to heightened rates of heart disease, certain cancers, and musculoskeletal disorders [17]. Recently, the impact of obesity on infectious diseases has also been confirmed [18, 19]. Therefore, these patients with high body mass index (BMI) might be at increased risk of COVID-19 and exhibit poor prognosis. However, few studies have explored the clinical findings of such patients. Therefore, this study aimed to report the epidemiological and clinical characteristics and outcomes of COVID-19 patients with high BMI and to investigate the association of high BMI with disease severity.

## Methods

### Study participants

The present retrospective cohort study focused on the epidemiological and, clinical characteristics and outcomes of imported COVID-19 patients with measured weight and height on admission; they were discharged before February 28, 2020, in designated hospitals of Zhejiang province, China. All patients with COVID-19 enrolled in this study were diagnosed according to the guidance formulated by the Chinese National Health Commission. This study was conducted in accordance with the guidelines of the Declaration of Helsinki and was approved by the Clinical Research Ethics Committee of the First Affiliated Hospital, College of Medicine, Zhejiang University.

### Data collection

The original data were collected by the Health Commission of Zhejiang Province. Herein, we utilized epidemiological, clinical, laboratory, radiological, therapeutic, and outcome data from the medical records of patients. At least two physicians independently reviewed the data according to standardized case report form. If clinical data was ambiguous or missing, we confirmed the details through directly contacting with the local medical staff or patients’ families. Laboratory confirmation of SARS-CoV-2 was test by real-time reverse transcription-polymerase chain reaction (RT-PCR) [20]. Laboratory data consisted of a complete blood count, coagulation function, blood biochemistry test (including electrolytes, renal and liver function), creatine kinase (CK), lactate dehydrogenase (LDH), C-reactive protein (CRP), and procalcitonin. Meanwhile, other respiratory pathogens, such as SARS- CoV, Middle east respiratory syndrome coronavirus (MERS-CoV), influenza A virus (H1N1, H3N2 and H7N9), influenza B virus, respiratory syncytial virus, parainfluenza virus, and adenovirus, were also identified. Treatments included antiviral therapy, antibiotic therapy, corticosteroid therapy, immunoglobulin therapy, mechanical ventilation, extracorporeal membrane oxygenation (ECMO) therapy, continuous renal replacement therapy (CRRT) and intensive care unit therapy.

### Definition

The severity of the disease was staged according to the diagnosis and treatment Protocol for COVID-19 Patients of Chinese (8th edition) [21], which was based on minor modification of the World Health Organization (WHO) standards. The subtypes were defined as follows. Mild type: clinical symptoms were mild, and there was no evidence of pneumonia in chest radiology; Moderate type: showing fever and respiratory symptoms with radiological findings of pneumonia; Severe type: meeting one of the following criteria: 1) Shortness of breath, respiratory frequency ≥ 30 breaths/minute; 2) resting blood oxygen saturation ≤ 93%; 3) partial pressure of arterial oxygen to fraction of inspired oxygen ratio < 300 mmHg; 4) radiographic progression, lung infiltration > 50% within 24–48 h; Critical type: meeting one of the following criteria: 1) severe respiratory failure requiring mechanical ventilation; 2) shock; 3) combined with other organ failure requiring intensive care treatment. The patient was discharged if the following criteria were fulfilled: 1) normalization of temperature for at least 3 days; 2) respiratory symptoms improved significantly; 3) lung infiltrates improved significantly in chest imaging; 4) SARS-CoV-2 RNA test negative in two consecutive respiratory specimens (sampling interval at least 24 h). The date of illness onset was defined as the day of presentation of clinical symptoms. The date of confirmed COVID-19 was defined as the day of laboratory confirmation. According to the Chinese consensus on overweight/obesity medical nutrition therapy (2016) [22], underweight is defined as BMI ≤ 18.5 kg/m^2^, overweight is defined as BMI ≥ 24 kg/m^2^, and obesity is defined as BMI ≥ 28 kg/m^2^. Glucose (Glu) refers specifically to fasting blood sugar 3.9–6.1 mmol/L.

### Statistical analysis

For continuous variable, mean (standard deviation, SD) was used for normally distributed data, while the median (interquartile range, IQR) was used if the data were skewed, followed by unpaired Student’s t-test and Mann–Whitney U test for comparison. Categorical variables were expressed as number (%) and compared by Pearson’s chi-squared test or Fisher’s exact test. Univariable logistic-regression analysis was utilized to identify the risk factors of Severe/Critical type pneumonia patients. All significant variables in univariable analysis was included in a multivariable logistic-regression model using the forward procedure: Wald method to identify independent predictors of severe/critical COVID-19 patients. The logistic-regression model was used to estimate the odds ratio (ORs) associated with BMI for the risk of progression to severe/critical illness after adjustment for pertinent variables. The ORs and 95% confidence intervals (CIs) of the progression to severe/critical illness in each subgroup were estimated, and their interactions tested. Likewise, multivariate logistic-regression analysis was also performed using high BMI as the dependent variable, including all significant variables in univariable analysis. A two-sided α < 0.05was considered statistically significant and SPSS (version 26.0) was used for all statistical analyses.

## Results

### Demographic and epidemiological characteristics

By February 28, 2020, a total of 565 COVID-19 patients were discharged in Zhejiang province, and BMI data of 455 patients were included in our retrospective study, 268 /455 were classified as underweight/normal weight group (Group A: BMI < 24), and the remaining187 comprised the overweight/obesity group (Group B: BMI ≥ 24), with corresponding median (IQR) BMI of 21.62(20.20–22.86) kg/m^2^ and 26.12(24.69–27.92) kg/m^2^, respectively (Table 1). The mean age in Group A was significantly lower than that in Group B (43.3 (15.7 y) vs. 46.3 (13.3 y), respectively, *P* = 0.028). The proportion of male patients was significantly higher in Group B than in Group A (63.6% vs. 36.2%, *P* < 0.001). However, no significant difference was noted in the percentage of exposure history in the two groups. A total of 20 (10.7%) patients in Group B were currently smoking, which was significantly higher than 14 (5.2%) patients in Group A(*P* = 0.029). The presence of any coexisting medical condition was also significantly higher in Group B than in Group A (43.9% vs 25.0%, *P* < 0.001), including the rate of hypertension (25.7% vs 9.7%, *P* < 0.001) and chronic liver disease (7.5% vs 3.0%, *P* = 0.028).

**Table 1:** Demographic and Epidemiological Characteristics of COVID-19 Patients with Different BMI

Characteristics	BMI < 24(Group A) (***N*** = 268)	BMI ≥ 24(Group B) (***N*** = 187)	***P*** value
**Age(years)**	43.3(15.7)	46.3(13.3)	**0.028**
**Sex**			**< 0.001**
men	97(36.2%)	119(63.6%)	
women	171(63.8%)	68(36.4%)	
**BMI (kg/m**^**2**^**)**	21.62(20.20–22.86)	26.12(24.69–27.92)	**< 0.001**
**Exposure history**			
Exposure to Wuhan	122(45.5%)	84(44.9%)	0.899
Contact with confirmed or suspected patients	130(48.5%)	76(40.6%)	0.097
Familial cluster	93(34.7%)	61(32.6%)	0.644
**Current smoking**	14(5.2%)	20(10.7%)	**0.029**
**Condition**			
Any	67(25.0%)	82(43.9%)	**< 0.001**
Hypertension	26(9.7%)	48(25.7%)	**< 0.001**
Diabetes	19(7.1%)	21(11.2%)	0.125
Heart disease	5(1.9%)	5(2.7%)	0.563
Chronic liver disease	8(3.0%)	14(7.5%)	**0.028**
Chronic renal disease	1(0.4%)	2(1.1%)	0.753
Cancer	3(1.1%)	3(1.6%)	0.977
COPD	0(0.0%)	1(0.5%)	0.411
Immunosuppression	0(0.0%)	1(0.5%)	0.411
**Signs and symptoms**			
Fever	213(79.5%)	164(87.7%)	**0.022**
Highest temperature(°C)			0.085
< 37.3	55(20.5%)	23(12.3%)	
37.3–38.0	92(34.3%)	80(42.8%)	
38.1–39.0	101(37.7%)	72(38.5%)	
> 39.0	20(7.5%)	12(6.4%)	
Cough	165(61.6%)	136(72.7%)	**0.013**
Sputum production	87(32.5%)	79(42.2%)	**0.033**
Sore throat	48(17.9%)	20(10.7%)	**0.034**
Nasal obstruction	20(7.5%)	5(2.7%)	**0.027**
Myalgia	32(11.9%)	20(10.7%)	0.681
Fatigue	58(21.6%)	35(18.7%)	0.787
GI symptoms^a^	36(13.4%)	30(16.0%)	0.437
Headache	20(7.5%)	23(12.3%)	0.083
**Incubation period (days)**	7(4–11) (*n* = 86)	7(4–10) (*n* = 46)	0.578
**Days from illness onset to first medical visit(days)**	2(1–5)	3(1–5)	0.143
**Days from illness onset to confirm the diagnosis(days)**	5(3–8)	5(3–8)	0.393
**Days from illness onset to first hospitalization(days)**	4(2–7)	4(3–8)	0.491
**Clinical Type**			**0.004**
Mild/ Moderate	249(92.9%)	158(84.5%)	
Severe/ Critical	19(7.1%)	29(15.5%)	

Data are presented as medians (interquartile ranges, IQR), n (%) and mean (SD)

GI symptoms^a^ include nausea, vomiting or diarrhea

The most common symptoms in the two groups were fever and cough. Group B showed significantly higher rate of fever (87.7% vs. 79.5%, *P* = 0.022), cough (72.7% vs. 61.6%, *P* = 0.013) and sputum production (42.2% vs. 32.5%, *P* = 0.033) than Group A. However, Group B showed a significantly lower rate of sore throat (10.7% vs. 17.9%, *P* = 0.034) and nasal obstruction (2.7% vs. 7.5%, *P* = 0.027). Furthermore, no significant differences were detected in the percentages of myalgia, fatigue, gastrointestinal (GI) symptoms, and headache. The incubation period, days from illness onset to first medical visit, days from illness onset to confirm the diagnosis and days from illness onset to first hospitalization between the two groups were not statistically significant. During the disease course, a significantly greater number of patients in Group B was diagnosed as severe/critical types than from Group A (15.5% vs. 7.1%, *P* = 0.004).

### Radiographic and laboratory findings

The radiographic and laboratory findings of the patients are shown in Table 2. On admission, Group B showed a significantly lower rate of neutropenia (12.8% vs. 23.9%, *P* = 0.003) and a higher level of hemoglobin (143.23 g/L vs. 133.52 g/L, *P* < 0.001). Furthermore, Group B showed significantly elevated level of alanine aminotransferase (ALT) (21.4% vs. 9.7%, *P* < 0.001) and aspartate aminotransferase (AST) (18.2% vs. 10.1%, *P* = 0.013). In addition, the median levels of ALT, AST, and total bilirubin (TB) in Group B were significantly higher than those in Group A (27 U/L vs. 18.4 U/L, *P* < 0.001; 27 U/L vs. 23 U/L, *P* < 0.001; 10.45 μmol/L vs. 8.90 μmol/L, *P* = 0.019, respectively). The median level of serum sodium in Group B was significantly lower (137.80 mmol/L vs. 138.60 mmol/L, *P* = 0.005) than in Group A, and a significantly higher rate of hyponatremia was observed in Group B (38.0% vs. 26.9%, *P* = 0.012). Group B showed a significantly higher median level of serum creatinine (Scr) (68.25 μmol/L vs 61.00 μmol/L, *P* < 0.001), CK (76.00 U/L vs 63.00 U/L, *P* = 0.004), LDH (212.50 U/L vs 202.00 U/L, *P* = 0.022), Glu (6.29 mmol/L vs 5.58 mmol/L, *P* < 0.001), and CRP (10.50 mg/L vs 6.10 mg/L, *P* < 0.001). According to the poor imaging findings during the disease course, the radiological findings showed that bilateral pneumonia was the most common in the two groups. In addition, increased prevalence of bilateral pneumonia and multiple mottling and ground-glass opacity in Group B were observed, although the difference was did not reach statistically significant (*P* = 0.053).

**Table 2:** Radiographic and Laboratory Findings of COVID-19 Patients with Different BMI

Characteristics	BMI < 24(Group A) (***N*** = 268)	BMI ≥ 24(Group B) (***N*** = 187)	***P*** value
**Blood routine**			
Leukocytes (×10^9^/L; normal range 4–10)	4.50(3.68–6.00)	4.95(3.91–6.04)	0.076
> 10 × 10^9^/L	5(1.9%)	3(1.6%)	1.000
< 4 × 10^9^/L	92(34.3%)	51(27.3%)	0.111
Neutrophils (× 10^9^/L; normal range 2–7)	2.78(2.02–3.96)	3.10(2.41–4.10)	0.286
> 7 × 10^9^/L	12(4.5%)	6(3.2%)	0.494
< 2 × 10^9^/L	64(23.9%)	24(12.8%)	**0.003**
Lymphocytes (×10^9^/L;normal range 0.8–4)	1.18(0.90–1. 60)	1.20(0.90–1.60)	0.880
< 0.8 × 10^9^/L	40(14.9%)	25(13.4%)	0.641
Hemoglobin (g/L, normal range: male 131–172, female 113–151)	133.52(16.11)	143.23(16.11)	**< 0.001**
Platelet (×10^9^/L; normal range:100–300)	191.00(145.00–228.00)	181.50(154.00–218.50)	0.528
< 100 × 10^9^/L	9(3.4%)	5(2.7%)	0.677
**Coagulation function**			
International normalized ration (normal range 0.85–1.15)	1.01(0.97–1.08)	1.01(0.97–1.08)	0.951
**Blood biochemistry**			
Albumin (g/L; normal range 40.0–55.0)	41.13(4.08)	41.61(4.12)	0.827
< 40.0 g/L	91(34.0%)	77(41.2%)	0.116
Alanine aminotransferase (U/L; normal range: male 9–50, female 7–40)	18.40(13.00–27.00)	27.00(18.25–42.75)	**< 0.001**
> 50 (male), > 40(female) U/L	26(9.7%)	40(21.4%)	**< 0.001**
Aspartate aminotransferase (U/L; normal range: male 15–40, female 15–35)	23.00(18.00–29.20)	27.00(21.00–35.38)	**< 0.001**
> 40 (male), > 35(female) U/L	27(10.1%)	34(18.2%)	**0.013**
Total bilirubin (umol/L; normal range 0–26.0)	8.90(6.00–12.75)	10.45(7.40–14.40)	**0.019**
> 26.0 umol/L	7(2.6%)	6(3.2%)	0.707
Serum potassium (mmol/L; normal range 3.5–5.3)	3.85(3.62–4.15)	3.80(3.60–4.08)	0.259
< 3.5 mmol/L	42(15.7%)	35(18.7%)	0.394
Serum sodium (mmol/L; normal range 137.0–147.0)	138.60(136.9–140.5)	137.80(135.6–139.7)	**0.005**
< 137.0 mmol/L	72(26.9%)	71(38.0%)	**0.012**
Blood urea nitrogen (mmol/L; normal range 3.1–8.0)	3.54(2.85–4.47)	3.75(2.99–4.60)	0.135
Serum creatinine (umol/L; normal range male: 57–97, female 41–73)	61.00(52.00–73.00)	68.25(56.00–80.20)	**< 0.001**
Creatine kinase (U/L; normal range: male40–200, female 50–130))	63.00(44.00–92.00)	76.00(51.50–99.00)	**0.004**
> 200 (male), > 130(female) U/L	29(10.8%)	23(12.3%)	0.626
Lactate dehydrogenase (U/L; normal range 120–250)	202.00(162.00–251.00)	212.50(170.50–276.00)	**0.022**
> 250 U/L	64(23.9%)	58(31.0%)	0.091
Glucose (mmol/L; normal range 3.9–6.1)	5.58(4.90–6.70)	6.29(5.36–7.85)	**< 0.001**
**Infection-related biomarkers**			
C-reactive protein (mg/L; normal range 0–8)	6.10(1.60–14.67)	10.50(4.00–24.20)	**< 0.001**
**Chest x-ray/CT findings**			0.053
Normal	21(7.8%)	11(5.9%)	
Unilateral pneumonia	61(22.8%)	25(13.4%)	
Bilateral pneumonia	117(43.7%)	94(50.3%)	
Multiple mottling and ground-glass opacity	69(25.7%)	57(30.5%)	

Data are presented as medians (interquartile ranges, IQR), n (%) and mean (SD)

### Treatment and outcomes

All 455 patients were isolated and treated in designated hospitals with supportive care and empiric medication (Table 3). The most common complication in the two groups was acute liver injury (26.7% vs 15.3%, respectively, *P* = 0.003). The rate of ARDS in Group B was higher than that in Group A (8.0% vs 2.6%, respectively, *P* = 0.003). The remaining complications did not show any statistical significance. Antiviral treatment was adopted for all of the patients except for one in Group A. These antiviral drugs included interferon-α sprays, arbidol hydrochloride capsules (2 tablets three times daily), lopinavir and ritonavir 2 tablets (500 mg) twice daily, orally.Several treatment options, including interferon-α sprays + lopinavir/ritonavir + arbidol triple combination treatment, interferon-α sprays + lopinavir/ritonavir combination treatment, interferon-α sprays + arbidol combination treatment, lopinavir/ritonavir + arbidol combination treatment and interferon-α sprays, arbidol, or lopinavir/ritonavir monotherapy, were available. The regimen of antiviral therapy during hospitalization and the number of days from illness onset to antiviral therapy did not differ significantly between the two groups. However, the median duration of antiviral therapy in Group B was significantly longer than in Group A (18 days vs. 16 days, *P* = 0.001). With respect to supportive treatment, the Group B showed significantly higher rates of antibiotic therapy (57.8% vs. 47.8%, *P* = 0.036), corticosteroid therapy (24.1% vs. 16.4%, *P* = 0.043), and immunoglobulin therapy (23.5% vs. 16.0%, *P* = 0.046), but no significant was detected in mechanical ventilation, ECMO therapy and intensive care unit therapy in two groups.

**Table 3:** Treatments and outcomes of COVID-19 patients with Different BMI

Characteristics	BMI < 24(Group A) (***N*** = 268)	BMI ≥ 24(Group B) (***N*** = 187)	***P*** value
**Complications**			
Acute respiratory distress syndrome	7(2.6%)	15(8.0%)	**0.008**
liver function abnormality	41(15.3%)	50(26.7%)	**0.003**
Acute kidney injury	0(0.0%)	0(0.0%)	
Shock	0(0.0%)	1(0.5%)	0.411
**Treatments**			
Antiviral treatment (n (%))	267(99.6%)	187(100%)	0.403
Days from illness onset to antiviral therapy (days)	5(3–8)	5(3–8)	0.926
Days of antiviral therapy	16(13–21)	18(14–23)	**0.001**
Antiviral regimen (days)			0.731
Interferon-α + Lopinavir/Ritonavir + arbidol	159(59.6%)	120(64.2%)	
Interferon-α + Lopinavir/Ritonavir	45(16.9%)	27(14.4%)	
Interferon-α + arbidol	19(7.1%)	13(7.0%)	
Lopinavir/Ritonavir+arbidol	33(12.4%)	18(9.6%)	
Others^a^	11(4.1%)	9(4.8%)	
Supportive treatment (n (%))			
Antibiotic therapy	128(47.8%)	108(57.8%)	**0.036**
Use of corticosteroid	44(16.4%)	45(24.1%)	**0.043**
Use of immunoglobulin	43(16.0%)	44(23.5%)	**0.046**
mechanical ventilation	2(0.7%)	6(3.2%)	0.109
CRRT	0(0.0%)	0(0.0%)	
ECMO	1(0.4%)	0(0.0%)	1.000
Admission to intensive care unit	1(0.4%)	2(1.1%)	0.753
**Clinical outcomes**			
Days from illness onset to SARS-CoV-2 RNA negative (days)	17(13–21)	18(14–25)	**0.015**
Days of hospitalization (days)	17(13–22)	19(15–24)	**0.003**
Days from illness onset to discharge (days)	21(17–26)	23(18–29)	**0.008**
Days of fever (days)	9(6–12)	9(6–12)	0.939
Days from first abnormal imaging findings to obvious absorption (days)	13(10–17)	13(10–16)	0.115

Data are presented as medians (interquartile ranges, IQR), n (%) and mean (SD)

Others^a^include interferon-α sprays, arbidol, and lopinavir/ritonavir monotherapy

All patients were discharged uneventfully. Group B showed significantly longer median of days from illness onset to SARS-CoV-2 negativity (18 days vs. 17 days, *P* = 0.015), days of hospitalization (19 days vs. 17 days, *P* = 0.003) and days from illness onset to discharge (23 days vs. 21 days, *P* = 0.008). However, the duration of fever and days from first abnormal imaging findings to obvious absorption were non-significant.

### Clinical characteristics of patients infected with SARS-CoV-2 with BMI ≥ 24

For further investigating the clinical characteristics of imported COVID-19 patients in the overweight/obesity group (Group B), patients with BMI ≥ 24 were divided into two groups according to clinical types (mild/ moderate group vs severe/critical group). As shown in Table S1, mild group and severe/critical group comprised of 158 and 29 patients, respectively. The severe/critical group showed significantly higher rates of exposure to Wuhan(69.0% vs. 40.5%, *P* = 0.005), coexisting medical condition(72.4% vs. 38.6%, *P* = 0.001), hypertension(41.4% vs. 22.8%, *P* = 0.035), chronic liver disease(27.6% vs. 3.8%, *P* < 0.001), fever(100.0% vs. 85.4%, *P* = 0.028), myalgia(24.1% vs. 8.2%, *P* = 0.011) and fatigue (34.5% vs. 17.7%, *P* = 0.039), and the highest temperature (*P* = 0.008) as compared to the mild group.

The radiographic and laboratory findings (Table S2), and low levels of lymphocytes and albumin were observed in the severe/critical group (*P* = 0.001 and *P* = 0.014, respectively), and higher rates of lymphopenia and hypoproteinemia were noted in the severe/critical group (31.0% vs. 10.1%, *P* = 0.002; 62.1% vs. 37.3%, *P* = 0.013, respectively). The severe/critical group showed significantly higher levels of serum sodium, Scr, CK, LDH, and CRP. An increased level of CK and LDH was observed in the severe/critical group as compared to the mild group (31.0% vs. 8.9%, *P* = 0.001; 58.6% vs. 25.9%, *P* < 0.001, respectively). Also, a significantly number of patients presented multiple mottling and ground-glass opacity in the severe/critical group than the mild group (*P* = 0.019).

Furthermore, the severe/critical group showed significantly higher rates of ARDS (44.8% vs. 1.3%, *P* < 0.001), antibiotic therapy (82.8% vs. 53.2%, *P* = 0.003), corticosteroid therapy (72.4% vs. 15.2%, *P* < 0.001), immunoglobulin therapy (69.0% vs. 15.2%, *P* < 0.001), mechanical ventilation(17.2% vs. 0.6%, *P* < 0.001) and admission to intensive care unit (6.9% vs. 0.0%, *P* = 0.023) than the mild group. Furthermore, the severe/critical group showed significantly longer median time as the number of days from illness onset to discharge (26 days vs. 23 days, *P* = 0.034), duration of fever (12 days vs. 9 days, *P* = 0.029) and from first abnormal imaging findings to obvious absorption (16 days vs. 13 days, *P* = 0.005) than the mild group. (Table S3).

### Risk factors predicted of progression to severe/critical illness

The cohort of this study comprised of 48 severe/critical COVID-19. In the univariable logistic regression of epidemiological, clinical and laboratory variables, 25 risk factors were significantly associated with severe/critical type (Table S4). Multivariable logistic regression demonstrated that BMI (per 1 kg/m^2^ increase) (OR: 1.168; 95%CI: 1.050–1.298, *P* = 0.004), exposure to Wuhan (OR: 4.214; 95%CI: 1.888–9.408, *P* < 0.001), any coexisting medical condition (OR: 3.885; 95%CI: 1.836–8.220, *P* < 0.001), highest temperature (OR: 2.521; 95%CI: 1.446–4.395, *P* = 0.001), CRP (OR: 1.025; 95%CI: 1.008–1.042, *P* = 0.003), and increased LDH (OR: 3.068; 95%CI: 1.423–6.615, *P* = 0.004) were independent risk factors associated with severe/critical illness (Table 4). For the sensitivity analysis, treating BMI as a categorical rather than continuous variable lead to similar results, showing a tendency for higher odds of progressing to severe/critically illness as higher BMI (OR: 1.20 per unit; 95% CI: 1.09–1.33; *P* = 0.0040). When adjusted for sex and age, the ratio of BMI > 28 compared to BMI < 24 was 3.70 (95% CI 1.57–8.71, *P* = 0.0046), and in the fully adjusted model, the odds of BMI as a clinical risk factor was 3.80 (95% CI 1.32–10.93, *P* = 0.032) (Table S5).

**Table 4:** Multivariable Analysis of Risk Factors associated for the Severe/Critical type COVID-19 patients

Risk Factors	Odds Ratio (95% CI)	***P*** value
**BMI (per 1 kg/m**^**2**^ **increase)**	1.168(1.050–1.298)	0.004
**Exposure to Wuhan**	4.214(1.888–9.408)	< 0.001
**Any coexisting medical condition**	3.885(1.836–8.220)	< 0.001
**Highest temperature**	2.521(1.446–4.395)	0.001
**CRP**	1.025(1.008–1.042)	0.003
**LDH (> 250 vs. ≤ 250)**	3.068(1.423–6.615)	0.004

### Risk factors associated with high BMI

This study comprised of 187 COVID-19 patients with BMI ≥ 24.According to Tables 1, 2 and 3, 26 risk factors were significantly associated with high BMI. Multivariable logistic regression showed that hemoglobin (OR: 1.031; 95%CI: 1.017–1.046, *P* < 0.001), ALT (OR: 1.038; 95%CI: 1.020–1.057, *P* < 0.001), CRP (OR: 1.024; 95%CI: 1.010–1.037, *P* < 0.001), and Scr (OR: 1.010; 95%CI: 1.002–1.019, *P* = 0.020) were independent associated factors with high BMI (Table 5).

**Table 5:** Multivariable Analysis of Risk Factors associated for COVID-19 patients with high BMI

Risk Factors	Odds Ratio (95% CI)	***P*** value
**Hemoglobin**	1.031(1.017–1.046)	< 0.001
**ALT**	1.038(1.020–1.057)	< 0.001
**Scr**	1.010(1.002–1.019)	0.02
**CRP**	1.024(1.010–1.037)	< 0.001

## Discussion

Several groups have discovered that overweight/obesity is correlated with the severity of illness and mortality in COVID-19 patients [23, 24]. However, those data are insufficient to reveal the clinical characteristics and outcomes of COVID-19 patients with overweight/obesity. Hence, we investigated the differences between patients with underweight/normal weight and patients with overweight/obesity in terms of clinical characteristics and the independent association of BMI with disease severity. According to these findings, patients with BMI ≥ 24 were mostly men, had smoking history, were severe/critical type, had acute/chronic liver injury and ARDS, longer hospitalization time, more number of days from illness onset to SARS-CoV-2 RNA negative, underwent prolonged anti-virus course, and had higher levels of ALT, AST, TB, Scr, CK, LDH, Glu, and CRP as compared to patients with BMI < 24. So, high BMI is an independent risk factor for severe/critical COVID-19 patients.

In this retrospective cohort study, 187 (41%) COVID-19 patients were overweight/obese. Commonly, overweight/obesity is closely related to increased morbidity and mortality to infectious diseases, and growing epidemiologic data can be cited in support of this conclusion [15, 18, 19, 25, 26].

The current study also proved that overweight/obese patients were highly vulnerable and had poor health. We also observed that such patients had several pre-existing diseases, such as hypertension and chronic liver disease. The initial clinical symptoms of pulmonary inflammation, including fever, cough and sputum production, were common in overweight/obese patients.

In addition to clinical findings, the laboratory results showed that obese patients also experienced worse illnesses. Some biochemical indicators, including ALT, AST, TB, Scr, CK, LDH, Glu, and CRP, indicating the injury of liver, kidney, myocardium, normal glucose regulation, more active inflammation, were significantly elevated in the blood of overweight/obese patients. These findings were consistent with the extensive distribution of ACE2 [9–12]. Moreover, because of the high level of these indicators, the risk of developing multiple organs dysfunction/failure syndrome in patients with overweight/obese is increased. This theory was supported by the higher rates of antibiotic therapy, corticosteroid therapy, immunoglobulin therapy and admission to the intensive care unit.

Although the reasons for increased infection risk of obesity are diverse, obesity-induced adipose tissue inflammation is considered a key player in the pathogenesis of infectious diseases, which might also play a major role in COVID-19. The adipose tissue secretes a series of immune regulators, termed as adipokines, such as leptin and adiponectin, which connect metabolism and immune system functions [27]. Obese individuals, because of fat accumulation and the dysregulation of adipokine secretion, exhibit disrupted lymphoid tissue integrity, changes in cytokine synthesis, reduced antigen response, and diminished function of natural killer cells, dendritic cells, and macrophages [26]. These factors promote obese individuals to produce inflammatory autoimmune responses and increase their sensitivity to infectious diseases.

Furthermore, excessive cytokines, such as interleukin (IL)-6, IL-8, monocyte chemoattractant protein-1 (MCP-1), and leptin produced by the dysfunctional adipocytes in obesity, leads to increased recruitment of macrophages. Interestingly, these cells produce abundant proinflammatory molecules, such as IL-1β, IL-6, IL-8, and MCP-1 [28]. Finally, the cytokine storm causes a hyperinflammatory reaction, which aggravates the COVID-19 infection. On the other hand, a recent study showed that the high expression of ACE2 in the adipose tissue not only plays a positive role in reversing the ACE1-induced damage but also promotes viral entrance [29]. Therefore, whether the mechanism of ACE2 on the adipose tissue or intensive cytokine storm produced by the dysfunctional adipocytes worsens COVID-19 outcomes is yet to be elucidated.

Previously, several studies revealed that obese hosts exhibit delayed and inactivated antiviral responses to influenza virus infection, and they recover poorly from the disease [30–32]. In addition, the efficacy of antiviral drugs was reduced in this population. Also, similar changes were observed in COVID-19 patients with overweight/obesity. In this study, compared to underweight/normal individuals, obese individuals had longer hospitalization, SARS-CoV-2 shedding, and anti-virus course and a higher proportion of severe/critical type. Notably, with the extension of the disease course, the risk of transmission to others increases, suggesting that obesity may play a critical role in SARS-CoV-2 transmission.

In addition, according to our data, most imported COVID-19 patients with BMI ≥ 24 in Zhejiang province were male patients, with significantly older age consistent with previous findings in the Chinese population [33]. Moreover, due to a high number of male patients, overweight/obese patients consisted largely of smokers.

We also compared the clinical characteristics and outcome of mild and severe/critical COVID-19 patients with overweight/obese. Firstly, severe/critical COVID-19 patients had been exposed to Wuhan. Secondly, differing from previous studies, the current results found that severe/critical patients had a significantly higher rate of myalgia and fatigue, which might result from an additional body burden due to excess fat. Therefore, we should be cautious about overweight/obese COVID-19 patients with these symptoms. The remaining clinical characteristics and outcome, including laboratory results, radiographic findings, treatment outcomes, were similar to those described in previous studies [34, 35].

The risk factors for severe/critical COVID-19 patients were calculated by multivariable logistic regression analysis. The total six factors are presented in Table 4. In summary, high BMI, exposure to Wuhan, any coexisting medical condition, highest temperature, CRP and increased LDH were independent risk factors for severe/critical COVID-19 patients. These findings indicated that overweight/obese COVID-19 patients had deteriorated health. A recent study from Zhejiang, China, also showed that in patients with metabolism-associated fatty liver disease, obesity increases the risk of severe COVID-19 illness [36].

Nevertheless, the current study has some limitations. First, weight and height should be measured in every patient. However, in our study, the weight and height of patients admitted with severe/ critical type could not be measured. Second, although the risk factors for severe/critical type of COVID-19 patients with overweight/obesity were identified, it still lacked of a prediction model for disease progression. Third, as the patients were only from Zhejiang province, it might be valuable when clinical features related to COVID-19 at a national level would be summarized. However, due to the limited sample size, we require further substantiation of the results in future studies.

## Conclusions

In summary, we reported the specific clinical characteristics and outcomes of imported COVID-19 patients with different BMIs. Contrasted with the imported COVID-19 patients with BMI < 24, high proportion of COVID-19 patients with BMI ≥ 24 in our study, especially those with elevated CRP and LDH, developed to severe type, with longer hospitalization duration and anti-virus course. In addition, high BMI was an independent risk factor for severe/critical COVID-19. Therefore, intensive attention should be paid to patients with high BMI to control the disease progression and the spread of the epidemic of COVID-19.

## Supplementary Information

**Additional file 1: Table S1**. Demographic and Epidemiological Characteristics of COVID-19 Patients with BMI ≥ 24. Data are presented as medians (interquartile ranges, IQR), n (%) and mean (SD).GI symptoms* include nausea, vomiting and diarrhea.

**Additional file 2: Table S2**. Radiographic and Laboratory Findings of COVID-19 Patients with BMI ≥ 24. Data are presented as medians (interquartile ranges, IQR), n (%) and mean (SD).

**Additional file 3: Table S3**. Treatments and outcomes of COVID-19 patients with BMI ≥ 24. Data are presented as medians (interquartile ranges, IQR), n (%) and mean (SD). Others* include interferon-α sprays, arbidol, and lopinavir/ritonavir monotherapy.

**Additional file 4: Table S4**. Univariable Analysis of Risk Factors associated for the Severe/Critical type COVID-19 patients.

**Additional file 5: Table S5**. Relationships between the BMI and Severe/ Critical using different models. Note: Model I adjusted for age, sex. Model II adjusted for age, sex, exposure to Wuhan, any coexisting medical condition, highest temperature, LDH, and C-reactive protein.

## Data Availability

The datasets used during the current study are available from the corresponding author on reasonable request.

## Abbreviations

COVID-19: Coronavirus disease 2019
SARS-CoV-2: Severe acute respiratory syndrome coronavirus 2
ARDS: Acute respiratory distress syndrome
ACE2: Angiotensin-converting enzyme 2
HIV: Human immunodeficiency virus
BMI: Body mass index
WHO: World health Organization
RT-PCR: Reverse transcription-polymerase chain reaction
MERS-CoV: Middle east respiratory syndrome coronavirus
SD: Standard deviation
IQR: Interquartile range
GI: Gastrointestinal
ALT: Alanine aminotransferase
AST: Aspartate aminotransferase
TB: Total bilirubin
Scr: Serum creatinine
CK: Creatine kinase
LDH: Lactate dehydrogenase
Glu: Glucose
CRP: C-reactive protein
ECMO: Extracorporeal membrane oxygenation
CRRT: Continuous renal replacement therapy
IL: Interleukin
MCP-1: Monocyte chemoattractant protein-1
