# Supplementary material for: High body mass index is a significant risk factor for the progression and prognosis of imported COVID-19: a multicenter, retrospective cohort study
Source: BMC Infect Dis. 2021 Feb 5;21:147. doi: 10.1186/s12879-021-05818-0 (PMC7863059; doi:10.1186/s12879-021-05818-0)
Supplement: Supplementary file 1 — Additional file 1: Table S1. Demographic and Epidemiological Characteristics of COVID-19 Patients with BMI ≥ 24. Data are presented as medians (interquartile ranges, IQR), n (%) and mean (SD).GI symptoms* include nausea, vomiting and diarrhea. [file 12879_2021_5818_MOESM1_ESM.docx]

**High** **body mass index is a significant risk factor for the progression and prognosis of imported COVID-19: a multicenter, retrospective cohort study**

**Journal title:** BMC Infectious Diseases.

**Huan Cai ^1†^ · Lisha Yang ^1†^ · Yingfeng Lu ^1†^· Shanyan Zhang ^1^ · Chanyuan Ye ^1^ · Xiaoli Zhang ^1^ · Guodong Yu ^1^ · Jueqing Gu ^1^ · Jiangshan Lian ^1^ · Shaorui Hao ^1^ · Jianhua Hu ^1^ · Yimin Zhang ^1^ · Ciliang Jin ^1^ ·Jifang Sheng ^1^ · Yida Yang ^1*^· Hongyu Jia ^1*^**

^1^State Key Laboratory for Diagnosis and Treatment of Infectious Diseases, National Clinical Research Center for Infectious Diseases, Collaborative Innovation Center for Diagnosis and Treatment of Infectious Diseases, Department of Infectious Diseases, The First Affiliated Hospital, College of Medicine, Zhejiang University, 79 Qingchun Rd., Hangzhou, China

*Correspondence: [jiahongyu@zju.edu.cn](mailto:jiahongyu@zju.edu.cn); [yidayang65@zju.edu.cn](mailto:yidayang65@zju.edu.cn)

^†^Huan Cai, Lisha Yang and Yingfeng Lu are co-first authors.

**Table S1 Demographic and Epidemiological Characteristics of COVID-19 Patients with BMI≥24**

| **Characteristics** | **Mild**  **(N=158)** | **Severe/Critical**  **(N=29)** | ***P* value** |
| --- | --- | --- | --- |
| **Age(years)** | 46.1(13.4) | 46.9(13.3) | 0.763 |
| **Sex** |  |  | 0.056 |
| men | 96(60.8%) | 23(79.3%) |  |
| women | 62(39.2%) | 6(20.7%) |  |
| **BMI (kg/m^2^)** | 26.02(24.74-27.69) | 27.55(24.51-29.18) | 0.126 |
| **Exposure history** |  |  |  |
| Exposure to Wuhan | 64(40.5%) | 20(69.0%) | **0.005** |
| Contact with confirmed  or suspected patients | 68(43.0%) | 8(27.6%) | 0.119 |
| Familial cluster | 54(34.2%) | 7(24.1%) | 0.289 |
| **Current smoking** | 16(10.1%) | 4(13.8%) | 0.557 |
| **Condition** |  |  |  |
| Any | 61(38.6%) | 21(72.4%) | **0.001** |
| Hypertension | 36(22.8%) | 12(41.4%) | **0.035** |
| Diabetes | 15(9.5%) | 6(20.7%) | 0.079 |
| Heart disease | 4(2.5%) | 1(3.4%) | 1.000 |
| Chronic liver disease | 6(3.8%) | 8(27.6%) | **<0.001** |
| Chronic renal disease | 1(0.6%) | 1(3.4%) | 0.709 |
| Cancer | 2(1.3%) | 1(3.4%) | 0.955 |
| COPD | 0(0.0%) | 1(3.4%) | 0.155 |
| Immunosuppression | 1(0.6%) | 0(0.0%) | 1.000 |
| **Signs and symptoms** |  |  |  |
| Fever | 135(85.4%) | 29(100.0%) | **0.028** |
| Highest temperature(°C) |  |  | **0.008** |
| <37.3 | 23(14.6%) | 0(0.0%) |  |
| 37.3–38.0 | 70(44.3%) | 10(34.5%) |  |
| 38.1–39.0 | 58(36.7%) | 14(48.3%) |  |
| >39.0 | 7(4.4%) | 5(17.2%) |  |
| Cough | 112(70.9%) | 24(82.8%) | 0.187 |
| Sputum production | 62(39.2%) | 17(58.6%) | 0.052 |
| Sore throat | 19(12.0%) | 1(3.4%) | 0.295 |
| Nasal obstruction | 5(3.2%) | 0(0.0%) | 1.000 |
| Myalgia | 13(8.2%) | 7(24.1%) | **0.011** |
| Fatigue | 28(17.7%) | 10(34.5%) | **0.039** |
| GI symptoms* | 23(14.6%) | 7(24.1%) | 0.196 |
| Headache | 17(10.8%) | 6(20.7%) | 0.134 |
| **Days from illness onset to first medical visit(days)** | 3(2-5) | 3(1-6) | 0.567 |
| **Days from illness onset to confirm the diagnosis(days)** | 5(3-8) | 7(3-10) | 0.445 |
| **Days from illness onset to first hospitalization(days)** | 4(3-8) | 6(2.5-8.5) | 0.424 |

Data are presented as medians (interquartile ranges, IQR), n (%) and mean (SD).

GI symptoms* include nausea, vomiting and diarrhea.
