# Supplementary material for: High body mass index is a significant risk factor for the progression and prognosis of imported COVID-19: a multicenter, retrospective cohort study
Source: BMC Infect Dis. 2021 Feb 5;21:147. doi: 10.1186/s12879-021-05818-0 (PMC7863059; doi:10.1186/s12879-021-05818-0)
Supplement: Supplementary file 2 — Additional file 2: Table S2. Radiographic and Laboratory Findings of COVID-19 Patients with BMI ≥ 24. Data are presented as medians (interquartile ranges, IQR), n (%) and mean (SD). [file 12879_2021_5818_MOESM2_ESM.docx]

**High** **body mass index is a significant risk factor for the progression and prognosis of imported COVID-19: a multicenter, retrospective cohort study**

**Journal title:** BMC Infectious Diseases.

**Huan Cai ^1†^ · Lisha Yang ^1†^ · Yingfeng Lu ^1†^· Shanyan Zhang ^1^ · Chanyuan Ye ^1^ · Xiaoli Zhang ^1^ · Guodong Yu ^1^ · Jueqing Gu ^1^ · Jiangshan Lian ^1^ · Shaorui Hao ^1^ · Jianhua Hu ^1^ · Yimin Zhang ^1^ · Ciliang Jin ^1^ ·Jifang Sheng ^1^ · Yida Yang ^1*^· Hongyu Jia ^1*^**

^1^State Key Laboratory for Diagnosis and Treatment of Infectious Diseases, National Clinical Research Center for Infectious Diseases, Collaborative Innovation Center for Diagnosis and Treatment of Infectious Diseases, Department of Infectious Diseases, The First Affiliated Hospital, College of Medicine, Zhejiang University, 79 Qingchun Rd., Hangzhou, China

*Correspondence: [jiahongyu@zju.edu.cn](mailto:jiahongyu@zju.edu.cn); [yidayang65@zju.edu.cn](mailto:yidayang65@zju.edu.cn)

^†^Huan Cai, Lisha Yang and Yingfeng Lu are co-first authors.

**Table S2 Radiographic and Laboratory Findings of COVID-19 Patients with BMI≥24**

| **Characteristics** | **Mild**  **(N=158)** | **Severe/Critical**  **(N=29)** | ***P* value** |
| --- | --- | --- | --- |
| **Blood routine** |  |  |  |
| Leukocytes  (×10^9^/L; normal range 4-10) | 5.14(3.99-6.05) | 4.35(3.78-5.43) | 0.353 |
| <4×10^9^/L | 41(25.9%) | 10(34.5%) | 0.343 |
| Neutrophils  (×10^9^/L; normal range 2-7) | 3.22(2.40-4.17) | 3.00(2.48-3.48) | 0.859 |
| <2×10^9^/L | 20(12.7%) | 4(13.8%) | 1.000 |
| Lymphocytes  (×10^9^/L; normal range 0.8-4) | 1.22(0.96-1. 64) | 0.96(0.74-1.26) | **0.001** |
| <0.8×10^9^/L | 16(10.1%) | 9(31.0%) | **0.002** |
| Hemoglobin  (g/L, normal range: male 131-172, female 113-151) | 142.37(16.30) | 142.86(14.00) | 0.882 |
| Platelet  (×10^9^/L; normal range:100-300) | 181.50(154.00-219.00) | 181.00(145.50-217.75) | 0.464 |
| <100×10^9^/L | 3(1.9%) | 2(6.9%) | 0.364 |
| **Coagulation function** |  |  |  |
| International normalized ration (normal range 0.85-1.15) | 1.01(0.96-1.08) | 1.02(0.99-1.10) | 0.412 |
| **Blood biochemistry** |  |  |  |
| Albumin  (g/L; normal range 40.0-55.0) | 41.53(4.26) | 39.43(3.74) | **0.014** |
| < 40.0 g/L | 59(37.3%) | 18(62.1%) | **0.013** |
| Alanine aminotransferase  (U/L; normal range: male 9-50, female 7-40) | 27.00(19.00-42.25) | 25.50(15.75-46.63) | 0.868 |
| >50 (male), >40(female) U/L | 33(20.9%) | 7(24.1%) | 0.695 |
| Aspartate aminotransferase  (U/L; normal range: male 15-40, female 15-35) | 27.00(21.00-35.13) | 28.00(20.25-39.30) | 0.521 |
| >40 (male), >35(female) U/L | 27(17.1%) | 7(24.1%) | 0.366 |
| Total bilirubin  (umol/L; normal range 0-26.0) | 10.45(7.36-14.40) | 10.70(7.30-15.08) | 0.716 |
| >26.0 umol/L | 4(2.5%) | 2(6.9%) | 0.514 |
| Serum potassium  (mmol/L; normal range 3.5-5.3) | 3.85(0.41) | 4.23(2.49) | 0.073 |
| <3.5 mmol/L | 28(17.7%) | 7(24.1%) | 0.415 |
| Serum sodium (mmol/L; normal range 137.0-147.0) | 132.56(24.85) | 137.27(3.46) | **0.016** |
| <137.0 mmol/L | 58(36.7%) | 13(44.8%) | 0.408 |
| Blood urea nitrogen  (mmol/L; normal range 3.1-8.0) | 3.70(2.98-4.60) | 4.01(3.05-4.68) | 0.190 |
| Serum creatinine  (umol/L; normal range male: 57-97, female 41-73) | 66.00(55.53-78.40) | 75.10(66.00-83.50) | **0.024** |
| Creatine kinase  (U/L; normal range: male40-200, female 50-130)) | 76.00(47.00-98.00) | 77.00(59.62-160.50) | **0.025** |
| >200 (male), >130(female) U/L | 14(8.9%) | 9(31.0%) | **0.001** |
| Lactate dehydrogenase  (U/L; normal range 120-250) | 204.00(169.50-269.50) | 263.10(205.25-318.00) | **0.003** |
| > 250 U/L | 41(25.9%) | 17(58.6%) | **<0.001** |
| Glucose  (mmol/L; normal range 3.9-6.1) | 6.25(5.32-7.80) | 6.30(5.44-8.29) | 0.343 |
| **Infection-related biomarkers** |  |  |  |
| C-reactive protein  (mg/L; normal range 0-8) | 8.75(3.48-21.74) | 21.25(15.76-40.67) | **<0.001** |
| **Chest x-ray/CT findings** |  |  | **0.019** |
| Normal | 11(7.0%) | 0(0.0%) |  |
| Unilateral pneumonia | 23(14.6%) | 2(6.9%) |  |
| Bilateral pneumonia | 83(52.5%) | 11(37.9%) |  |
| Multiple mottling and  ground-glass opacity | 41(25.9%) | 16(55.2%) |  |

Data are presented as medians (interquartile ranges, IQR), n (%) and mean (SD).
