# Supplementary material for: High body mass index is a significant risk factor for the progression and prognosis of imported COVID-19: a multicenter, retrospective cohort study
Source: BMC Infect Dis. 2021 Feb 5;21:147. doi: 10.1186/s12879-021-05818-0 (PMC7863059; doi:10.1186/s12879-021-05818-0)
Supplement: Supplementary file 3 — Additional file 3: Table S3. Treatments and outcomes of COVID-19 patients with BMI ≥ 24. Data are presented as medians (interquartile ranges, IQR), n (%) and mean (SD). Others* include interferon-α sprays, arbidol, and lopinavir/ritonavir monotherapy. [file 12879_2021_5818_MOESM3_ESM.docx]

**High** **body mass index is a significant risk factor for the progression and prognosis of imported COVID-19: a multicenter, retrospective cohort study**

**Journal title:** BMC Infectious Diseases.

**Huan Cai ^1†^ · Lisha Yang ^1†^ · Yingfeng Lu ^1†^· Shanyan Zhang ^1^ · Chanyuan Ye ^1^ · Xiaoli Zhang ^1^ · Guodong Yu ^1^ · Jueqing Gu ^1^ · Jiangshan Lian ^1^ · Shaorui Hao ^1^ · Jianhua Hu ^1^ · Yimin Zhang ^1^ · Ciliang Jin ^1^ ·Jifang Sheng ^1^ · Yida Yang ^1*^· Hongyu Jia ^1*^**

^1^State Key Laboratory for Diagnosis and Treatment of Infectious Diseases, National Clinical Research Center for Infectious Diseases, Collaborative Innovation Center for Diagnosis and Treatment of Infectious Diseases, Department of Infectious Diseases, The First Affiliated Hospital, College of Medicine, Zhejiang University, 79 Qingchun Rd., Hangzhou, China

*Correspondence: [jiahongyu@zju.edu.cn](mailto:jiahongyu@zju.edu.cn); [yidayang65@zju.edu.cn](mailto:yidayang65@zju.edu.cn)

^†^Huan Cai, Lisha Yang and Yingfeng Lu are co-first authors.

**Table S3 Treatments and outcomes of COVID-19 patients with BMI≥24**

| **Characteristic** | **Mild**  **(N=158)** | **Severe/Critical**  **(N=29)** | ***P* value** |
| --- | --- | --- | --- |
| **Complications** |  |  |  |
| Acute respiratory distress syndrome | 2(1.3%) | 13(44.8%) | **<0.001** |
| liver function abnormality | 42(26.6%) | 8(27.6%) | 0.911 |
| Acute kidney injury | 0(0.0%) | 0(0.0%) |  |
| Shock | 0(0.0%) | 1(3.4%) | 0.155 |
| **Treatments** |  |  |  |
| Antiviral treatment (n (%)) | 187(100%) | 29(100%) |  |
| Days from illness onset to  antiviral therapy | 5(3-8) | 5(3-9.5) | 0.670 |
| Days of antiviral therapy | 18(14-23) | 21(17.5-25) | **0.029** |
| Antiviral regimen |  |  | 0.347 |
| Interferon-α +Lopinavir/Ritonavir +arbidol | 100(63.3%) | 20(69.0%) |  |
| Interferon-α +Lopinavir/Ritonavir | 25(15.8%) | 2(6.9%) |  |
| Interferon-α+arbidol | 12(7.6%) | 1(3.4%) |  |
| Lopinavir/Ritonavir +arbidol | 12(7.6%) | 6(20.7%) |  |
| Others* | 9(4.8%) | 0(0.0%) |  |
| **Supportive treatments** (n (%)) |  |  |  |
| Antibiotic therapy | 84(53.2%) | 24(82.8%) | **0.003** |
| Use of corticosteroid | 24(15.2%) | 21(72.4%) | **<0.001** |
| Use of immunoglobulin | 24(15.2%) | 20(69.0%) | **<0.001** |
| mechanical ventilation | 1(0.6%) | 5(17.2%) | **<0.001** |
| CRRT | 0(0.0%) | 0(0.0%) |  |
| ECMO | 0(0.0%) | 0(0.0%) |  |
| Admission to intensive care unit | 0(0.0%) | 2(6.9%) | **0.023** |
| **Clinical outcomes** |  |  |  |
| Days from illness onset to  SARS-CoV-2RNA negative (days) | 18(14-25) | 20(16.5-27) | 0.078 |
| Days of hospitalization (days) | 18(14-24) | 21(17.5-26) | 0.067 |
| Days from illness onset to discharge(days) | 23(19-29) | 26(21-31) | **0.034** |
| Days of fever(days) | 9(6-12) | 12(7.5-13.5) | **0.029** |
| Days from first abnormal imaging findings to obvious absorption(days) | 13(10-16) | 16(12.5-19.5) | **0.005** |

Data are presented as medians (interquartile ranges, IQR), n (%) and mean (SD).

Others* include interferon-α sprays, arbidol, and lopinavir/ritonavir monotherapy.
