# Supplementary material for: High body mass index is a significant risk factor for the progression and prognosis of imported COVID-19: a multicenter, retrospective cohort study
Source: BMC Infect Dis. 2021 Feb 5;21:147. doi: 10.1186/s12879-021-05818-0 (PMC7863059; doi:10.1186/s12879-021-05818-0)
Supplement: Supplementary file 5 — Additional file 5: Table S5. Relationships between the BMI and Severe/ Critical using different models. Note: Model I adjusted for age, sex. Model II adjusted for age, sex, exposure to Wuhan, any coexisting medical condition, highest temperature, LDH, and C-reactive protein. [file 12879_2021_5818_MOESM5_ESM.doc]

**High** **body mass index is a significant risk factor for the progression and prognosis of imported COVID-19: a multicenter, retrospective cohort study**

**Journal title:** BMC Infectious Diseases.

**Huan Cai ^1†^ · Lisha Yang ^1†^ · Yingfeng Lu ^1†^· Shanyan Zhang ^1^ · Chanyuan Ye ^1^ · Xiaoli Zhang ^1^ · Guodong Yu ^1^ · Jueqing Gu ^1^ · Jiangshan Lian ^1^ · Shaorui Hao ^1^ · Jianhua Hu ^1^ · Yimin Zhang ^1^ · Ciliang Jin ^1^ ·Jifang Sheng ^1^ · Yida Yang ^1*^· Hongyu Jia ^1*^**

^1^State Key Laboratory for Diagnosis and Treatment of Infectious Diseases, National Clinical Research Center for Infectious Diseases, Collaborative Innovation Center for Diagnosis and Treatment of Infectious Diseases, Department of Infectious Diseases, The First Affiliated Hospital, College of Medicine, Zhejiang University, 79 Qingchun Rd., Hangzhou, China

*Correspondence: [jiahongyu@zju.edu.cn](mailto:jiahongyu@zju.edu.cn); [yidayang65@zju.edu.cn](mailto:yidayang65@zju.edu.cn)

^†^Huan Cai, Lisha Yang and Yingfeng Lu are co-first authors.

**Table S5 Relationships between the BMI and Severe/ Critical using different models**

| BMI (kg/m2) | Total, n | | Severe/ Critical,  n (%) | OR (95% CI) | | |
| --- | --- | --- | --- | --- | --- | --- |
|  |  |  |  | Non-adjusted | Adjust I | Adjust II |
| BMI <24 | | 268 | 19 (7.1%) | Reference | Reference | Reference |
| BMI ≥24, <28 | | 139 | 17 (12.2%) | 1.83 (0.92- 3.64) | 1.51 (0.75- 3.07) | 1.11 (0.47- 2.63) |
| BMI ≥28 | | 48 | 12 (25.0%) | 4.37 (1.96-9.75) | 3.70 (1.57- 8.71) | 3.80 (1.32- 10.93) |
| P for trend | | |  | 0.0040 | 0.0046 | 0.0320 |
| Increase per unit | | | | 1.20 (1.09- 1.33) | 1.17 (1.05- 1.31) | 1.16 (1.01- 1.33) |

Note: Model I adjusted for age, sex. Model II adjusted for age, sex, exposure to Wuhan, any coexisting medical condition, highest temperature, LDH, and C-reactive protein.
